# Stealthy Vehicle Adversarial Camouflage Texture Generation Based on Neural Style Transfer

**DOI:** 10.3390/e26110903

**Published:** 2024-10-24

**Authors:** Wei Cai, Xingyu Di, Xin Wang, Weijie Gao, Haoran Jia

**Affiliations:** The Third Faculty of Xi’an Research Institute of High Technology, Xi’an 710064, China; xhtu807@outlook.com (W.C.); wangxin9550@outlook.com (X.W.); gaoweijie331@outlook.com (W.G.); rq159357@outlook.com (H.J.)

**Keywords:** physical attack, neural style transfer, stealthy adversarial attack, white-box attack, object detection

## Abstract

Adversarial attacks that mislead deep neural networks (DNNs) into making incorrect predictions can also be implemented in the physical world. However, most of the existing adversarial camouflage textures that attack object detection models only consider the effectiveness of the attack, ignoring the stealthiness of adversarial attacks, resulting in the generated adversarial camouflage textures appearing abrupt to human observers. To address this issue, we propose a style transfer module added to an adversarial texture generation framework. By calculating the style loss between the texture and the specified style image, the adversarial texture generated by the model is guided to have good stealthiness and is not easily detected by DNNs and human observers in specific scenes. Experiments have shown that in both the digital and physical worlds, the vehicle full coverage adversarial camouflage texture we create has good stealthiness and can effectively fool advanced DNN object detectors while evading human observers in specific scenes.

## 1. Introduction

DNNs have garnered extensive attention due to their exceptional performance across a range of tasks. In the realm of visual tasks, object detection necessitates precise classification and positioning, forming the cornerstone for other visual tasks such as instance segmentation and object tracking [1]. Meanwhile, it finds wide-ranging applications in fault detection [2] and autonomous driving [3]. However, well-trained DNNs are susceptible to adversarial examples, which exploit the vulnerabilities of DNNs and cause DNNs to output incorrect results when given adversarial examples, for example, using differential privacy to generate adversarial examples to attack deep learning-based Android malware detection systems [4]. Recent research indicates that real-world adversarial examples can effectively attack DNNs deployed in physical environments, posing a greater threat than digital attacks. This is because attackers only need to manipulate the environment, objects, or sensing devices outside the model in the real world to launch an attack without altering the digital images or video input to the DNN, thus endangering information security and property safety. However, physical adversarial attacks in real-world scenes present greater challenges compared to digital attacks that manipulate pixel-level digital images. This is because while the target of digital attacks is to perturb images in a manner imperceptible to the human eye, subtle perturbations are hindered by various factors such as spatial distortion, changes in lighting, and limitations of the camera during image capture. Consequently, in the physical domain, adversarial examples intended for attack purposes amplify the amplitude of perturbations to ensure the successful execution of the attack. As a result, these generated adversarial examples become more conspicuous to human observers and are easily identifiable.

Most existing attack methods primarily target the digital domain, seeking to introduce perturbations globally or locally to the example. Furthermore, current research on physical adversarial attacks predominantly emphasizes attack effectiveness, with limited consideration given to the stealthiness of an attack. In existing research on stealthy attacks, various approaches exist: SC-PCA [5] and NPA [6] utilize generative adversarial networks (GANs) to confine perturbations within a specific range when generating adversarial patches; AdvCam [7] and Rust-Style Patch [8] employ neural style transfer (NST) technology to blend adversarial examples with natural styles, enabling them to appear inconspicuous in front of cameras without detection by human observers; LAP [9] and DAS [10] produce adversarial examples by constraining similarity between the input image’s features and those of the original image. Currently, these stealthy adversarial example-generation methods are limited to flat patches and can only perform attacks at specific angles. Even if DAS and SC-PCA apply adversarial patches to the vehicle at multiple angles, the attack angle is still limited and does not cover the entire body paint with adversarial camouflage. Existing full-coverage adversarial camouflage methods such as FCA [11], MFA [12], CAC [13], DTA [14], and ACTIVE [15] only focus on the attacking nature of adversarial camouflage but do not consider its stealthiness. In response to these challenges, we propose a method for generating full-coverage vehicle camouflage textures based on NST. The main contributions to this article are as follows:

1. Our proposed method combines NST with vehicle full coverage adversarial camouflage texture generation to generate stealthy adversarial examples with specific image styles that cannot be detected by the victim model in specific scenes;
2. We train textures on neural networks based on neural rendering and collect test images in modeling and rendering software based on traditional physical rendering to make the test data closer to real physical scenes and capture and collect data in the real physical world;
3. We fix the flaws in the dataset of other people’s previous papers, making the digital simulation effect closer to the real physical world and reducing the gap in the transformation of adversarial camouflage textures from the digital domain to the physical domain.

## 2. Related Work

### 2.1. Neural Style Transfer

Style transfer technology integrates the content of one image with the style of another while preserving the original image’s content and texture features. Traditional style transfer techniques are mainly used to blend artistic styles, employing key methods such as texture synthesis and non-photorealistic rendering. NST extracts content and style information from different layers of a neural network and then recombines them to produce a new image, significantly enhancing the effectiveness of the transfer. This process is mainly categorized into image iteration-based methods and model iteration-based methods [16]. And NST has extended its reach beyond the domain of artistic style to encompass disciplines such as medicine, industry, and literature [17]. Gatys et al. [18] used the VGG19 neural network model to extract content information and style information for the first time and then iteratively generated stylized images from noisy images. LI et al. [19] proposed replacing the Gram matrix used for extracting style in the method of [18] with a Markov random field. Johnson et al. [20] proposed a style transfer model based on model iteration, where a feedforward neural network model was trained to transform images into oriented styles. Din et al. [21] integrated GAN with style transfer techniques to construct a cross-modal facial synthesis network. Wen et al. [22] introduced the CAP-VSTNet, which employs invertible residual networks and Cholesky decomposition-based unbiased linear transformation modules for style transfer in the feature space, maintaining superior content affinity. Wang et al. [23] used diffusion models to achieve style transfer, achieving more explainable and controllable content-style decoupling, which improved the quality of style transfer.

### 2.2. Stealthy Physical Adversarial Examples

Current adversarial examples are designed to introduce subtle perturbations that are imperceptible to human observers but can mislead DNNs. However, capturing these subtle perturbations with physical world cameras is challenging, making it difficult to replicate such attacks in the real world. Physical-world attacks often require large or unbounded perturbations, which may be easily detected by observers, thus limiting their effectiveness in executing successful attacks in the physical domain, as demonstrated by adversarial-YOLO [24] and adversarial bulb [25].

To tackle this challenge, researchers have explored a range of methodologies. The adversarial T-shirt [26] leverages thin plate spline interpolation to integrate adversarial examples into deformable objects, such as clothing, rather than applying adversarial patches onto rigid surfaces. AdvCam generates unrestricted-size adversarial examples and computes style loss to incorporate adversarial perturbations into plausible style designs (e.g., rust on road signs). DAS and SC-PCA introduce perturbations to patterns in order to evade human visual attention and thwart the accurate detection of vehicles by DNNs; RFLA [27] is capable of launching attacks in diverse environments using natural sunlight and artificial flashlights.

Our method meticulously combines the innovative ideas from the aforementioned works, incorporating various techniques, such as AdvCam incorporating adversarial perturbations into specific styles to generate adversarial examples, Adversarial T-shirt attaching adversarial samples to objects in appropriate forms instead of placing a whole plane in front of the target, and DAS using neural renderers to train adversarial examples. By integrating these pioneering concepts, our method achieves a balanced and potent approach to adversarial attacks, capable of evading detection while maintaining high efficacy.

### 2.3. Camouflage Effect Evaluation

To address the absence of quantitative metrics for assessing the stealthiness of adversarial examples, researchers have proposed benchmark tests and evaluation methods to quantify the visual naturalness of real-world adversarial attacks. LI et al. [28] introduced the PAN dataset—a dataset designed to investigate the naturalness of physical world attacks through human ratings and gaze tracking, and they assessed human vision rationality by gathering eye-tracking data from 126 volunteers. They also suggested dual prior alignment to incorporate human behavior into model inference, thus achieving improved results and generalization capability.

A more widely applicable method for covert assessment involves utilizing image quality evaluation techniques, such as SSIM and PSNR, to gauge the perceptual variance between the unaltered and altered images and to quantitatively evaluate the stealthiness of adversarial examples in the scene by comparing the scores of different attack methods. The subsequent sections will provide an overview of these image quality evaluation techniques, which will be employed for comparative analysis in assessing stealth effects.

#### 2.3.1. SSIM

The Structural Similarity Index Measure (SSIM) is an index used to evaluate image similarity, which takes into account not only the brightness, contrast, and structure of an image but also the perceptual characteristics of the image as perceived by the human eye. SSIM calculates the similarity between two images by comparing the similarity between their pixels, thus measuring the degree of similarity between them. SSIM’s calculation method is based on the local regions of an image, which can more accurately evaluate the quality of an image. Compared with other image similarity indicators, SSIM has better stability and reliability and is widely used in image processing, image compression, image enhancement, etc. The SSIM calculation can be expressed using the following formula.

(1) $$l(x,y)=\frac{2\mu_{x}\mu_{y}+C_{1}}{\mu_{x}^{2}\mu_{y}^{2}+C_{1}},$$

(2) $$c(x,y)=\frac{2\sigma_{x}\sigma_{y}+C_{2}}{\sigma_{x}^{2}\sigma_{y}^{2}+C_{2}},$$

(3) $$s(x,y)=\frac{2\sigma_{xy}+C_{3}}{\sigma_{x}\sigma_{y}+C_{3}},$$

(4) $$\mathrm{SSIM}(x,y)=l{\left(x,y\right)}^{\alpha }\cdot c{\left(x,y\right)}^{\beta }\cdot s{\left(x,y\right)}^{\gamma },$$

specifically, $\mu_{x}$ and $\mu_{y}$ are the average of the sum of all pixel values in images x and y, l(x,y) measures the brightness difference between images x and y; $\sigma_{x}$ and $\sigma_{y}$ are the standard deviation of images x and y, c(x,y) measures the contrast difference between images x and y; $\sigma_{xy}$ represents the covariance between images x and y, s(x,y) represents the structural difference between the two images; $C_{1}$, $C_{2}$, $C_{3}$ and are non-zero constants to prevent pixel values from being zero; α, β and γ are the weights controlling the three indicators.

#### 2.3.2. PSNR

The peak signal-to-noise ratio (PSNR) is utilized to assess image quality by computing the peak signal-to-noise ratio between the original and processed images. A higher PSNR value indicates superior image quality. We will employ PSNR to discern disparities between adversarial examples with adversarial camouflage texture and the original scene images.

(5) $$MSE=\frac{1}{mn}\sum_{i=0}^{m-1}\sum_{j=0}^{n-1}{\left[I(i,j)-K(i,j)\right]}^{2},$$

(6) $$PSNR=10\cdot \log_{10}(\frac{MAX_{I}^{2}}{MSE}),$$

specifically, *MAX* denotes the maximum pixel value in the image (typically 255), *MSE* stands for mean squared error, *I* represents the clean image (in this paper, the original scene image), and *K* denotes the noisy image (in this paper, the scene image with adversarial examples).

#### 2.3.3. LPIPS

Learning Perceptual Image Patch Similarity (LPIPS) measures the similarity of image patches using deep features, which allows for a more accurate assessment of image similarity than traditional image quality metrics [29]. Laidlaw et al. [30] found that LPIPS has a good correlation with naturalness. A higher LPIPS value indicates a more significant perceived difference between the two images. Unlike traditional image quality metrics, LPIPS compares images at the deep feature level, which better aligns with the perceptual characteristics of the human visual system. LPIPS is not based on mathematical formulas but rather is implemented using DNNs.

## 3. Methods

**Figure 1:**
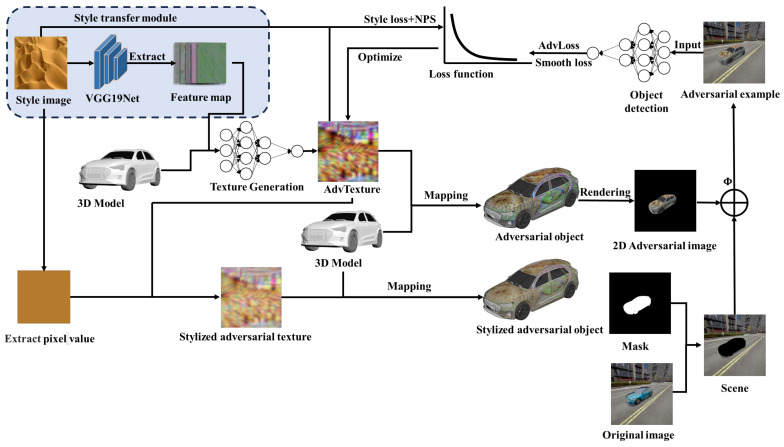
Pipeline of vehicle full coverage stealth adversarial camouflage texture generation method. Our training set comes from vehicle images collected from different angles in Carla, which were publicly released by DAS. Initially, the VGG19 network is employed to extract the feature maps of the specified style image, and then it is input together with the three-dimensional (3D) model into the adversarial texture generation model. The generated texture (initially random noise) is then applied to the surface of the vehicle according to the specified face document. Covered with an adversarial texture, vehicles are rendered as two-dimensional (2D) images using a differentiable neural mesh renderer [31] (NMR) according to the position and orientation information of the vehicle and camera in the original image and then synthesized into the scene of the original image through a binary mask m ($m\in {\mathbb{R}}^{H\times W\times 1}$) to create an adversarial example $x_{adv}$ ($x_{adv}\in {\mathbb{R}}^{H\times W\times 3}$). A physical transformation function is employed to bridge the gap between digital and physical domains. The resulting adversarial examples are input into an object detection model to compute adversarial loss, smoothing loss, and style loss for iterative optimization of texture information, ultimately generating an adversarial camouflage texture with the style of image texture feature. In order to minimize the color disparity between the adversarial camouflage texture and the image style, we extract the pixel value from the style image and integrate them with the adversarial camouflage texture to create a stealthy adversarial camouflage texture.

We improved the existing framework and proposed a method for generating full coverage of adversarial camouflage texture, mainly by utilizing NST to guide the model in generating texture patterns with the features of style images. Figure 1 shows the framework of our method.

Our method accesses the structure, weights, and parameters of the YOLOv3 model in a white-box setting. It optimizes the texture by using the AdamW optimizer. The generation of adversarial camouflage texture is set as an optimization problem, where the texture is optimized by optimizing the loss function so that the vehicle covered by the adversarial texture cannot be correctly detected by the YOLOv3 object detection model. The objective function is represented as follows:

(7) $$T_{adv}^{*}=\underset{T_{adv}}{\arg\max}\;J(F(\Phi (R(M,T_{adv};e,\theta ;\theta_{f}),Y),$$

where $T_{adv}^{*}$ represents the optimal adversarial texture; J represents the loss function utilized to quantify the disparity between the predicted output and the ground truth; F is a target detection model with parameters $\theta_{f}$; Φ is the transformation function that converts the rendered 2D image into various scenes; e and θ denote the position and orientation information of the vehicle and camera, while Y signifies the ground truth label set of dataset X.

### 3.1. Style Transfer Module

Our proposed style transfer module commences with the specification of a style image as the reference, followed by loading a pre-trained VGG19 model for extracting image feature representations. Subsequently, a sequence of feature maps is obtained through convolutional layers, pooling layers, and activation functions. The next step involves calculating the Gram matrix between the generated texture and the style image’s feature representation at a specified layer. Finally, we compute the style loss using MSE and integrate it into the overall loss calculation to facilitate gradual convergence of the generated texture towards the stylistic characteristics of the specified style image.

### 3.2. Loss Function

The loss function consists of three parts: smooth loss, adversarial loss, and style loss. The overall loss function is formulated as follows:

(8) $${ℒ}_{total}={ℒ}_{smooth}+{ℒ}_{adv}+{ℒ}_{style}+{ℒ}_{NPS},$$

the adversarial loss includes three losses defined in the object detection model: bounding box loss, classification loss, and objectness loss. The definition of these loss functions in object detection is to enable the model to correctly detect the target, while in adversarial loss, the definition of these loss terms is the opposite for object detection models: object detection loss can be understood as the model’s goal of minimizing classification and confidence losses, while adversarial loss is to maximize these losses, that is, to make the model produce more erroneous classification, bounding box, and confidence predictions. The formula is expressed as follows:

(9) $${ℒ}_{adv}=\alpha \prime {ℒ}_{adv}^{box}+\beta \prime {ℒ}_{adv}^{obj}+\gamma \prime {ℒ}_{adv}^{cls},$$

the weights for each loss item, denoted as α′, β′ and γ′, are specified in the YOLOv3 code as follows: α′ = 0.05, β′ = 1.0, and γ′ = 0.5.

#### 3.2.1. Style Loss

Our style loss is measured by calculating the style difference between the generated texture and the specified style image, by calculating the MSE between the Gram matrices of the generated images and the specified style images at different layers during training, and then the weighted sum is taken to obtain the final style loss value. The model can learn to generate textures with specified styles. The formula is expressed as follows:

(10) $${ℒ}_{style}=\sum_{l\in L}w_{l}\cdot \frac{1}{C_{l}\cdot H_{l}\cdot W_{l}}\cdot ||G(T_{l})-G(S_{l}){||}_{F}^{2},$$

where $w_{l}$ denotes the weight of layer l, L denotes the set of specifically chosen convolutional layers, $C_{l}$, $W_{l}$, and $H_{l}$ denote the number of channels, height, and width of the layer, respectively. Their product represents the sum of all features across all channels, G(⋅) represents the Gram matrix, and $||\cdot |{|}_{F}$ represents the Frobenius norm, which is defined as the square root of the sum of squares of all elements in the matrix.

#### 3.2.2. Adversarial Loss

The classification loss comprises three components, structured in a manner analogous to the loss terms in the object detection model, with inverted implications.

Classification Loss: The classification loss is utilized to gauge the precision of the object detection model in predicting the object category, and the classification performance is assessed by contrasting the disparity between the model’s predicted category and the true category. Our approach employs binary cross-entropy loss, wherein a positive-predicted label value is set as 0, and a negative-predicted label value is set as 1, leading to disregard of correct classification information by the model and acquisition of erroneous classification information, thereby tending to optimize texture towards misclassification. The formula can be expressed as follows:

(11) $${ℒ}_{adv}^{cls}=\frac{1}{N}\sum_{1=1}^{N}-[y_{i}\cdot \log(p_{i})+(1-y_{i})\cdot \log(1-p_{i})],$$

where y represents the ground truth label value, where the positive label value is 0 and the negative label value is 1. $p_{i}$ represents the probability of the model predicting the classification. Therefore, when the prediction is for a positive class ($y_{i}=0$), the smaller the predicted probability, the smaller the value of the loss function. When the prediction is for a negative class ($y_{i}=1$), the larger the predicted probability, the smaller the value of the loss function.

Bounding Box Regression Loss: The bounding box regression loss is used in object detection algorithms to measure the difference between the predicted box and the ground truth box. In object detection tasks, the bounding box loss evaluates the overlap between the predicted box and the ground truth box by calculating their Intersection over Union (IoU). During adversarial loss computation, minimizing IoU between the predicted box and the true box adjusts the model towards incorrectly predicted bounding boxes. The formula is expressed as follows:

(12) $${ℒ}_{adv}^{box}=\sum_{i=1}^{N}IoU(b_{i},b_{i}^{gt}),$$

specifically, N represents the number of predicted boxes output by the YOLO model during detection (N=3), and $b_{i}$ and $b_{i}^{gt}$ are the model’s predicted results for the i-th scale of the target and the corresponding ground truth.

Objectness Loss: The confidence loss indicates the certainty that the identified box contains an object. We have adopted the confidence loss setting in FCA and selected the target confidence score as our ${ℒ}_{adv}^{obj}$.

#### 3.2.3. Smooth Loss

The smooth loss is designed to make the boundary-contrasting perturbations less abrupt and smoother, reducing the difference between adjacent pixels in the generated adversarial texture, ensuring the smoothness and continuity of the texture; we quote the total variation proposed by Mahendran [32] et al. to define the smooth loss, the formula is expressed as follows:

(13) $${ℒ}_{smooth}=\sum_{i,j}(x_{i,j}-x_{i+1,j}{)}^{2}+\sum_{i,j}(x_{i,j}-x_{i,j+1}{)}^{2},$$

$x_{i,j}$ denotes the pixel value at the coordinate (i,j) of the adversarial example, which indicates the luminance or color information of this pixel.

#### 3.2.4. NPS

To ensure that the colors used in the generated adversarial camouflage texture can be printed by the printer, we adopt the Non-Printability Score (NPS) employed by Sharif et al. [33] as a loss term to minimize the adversarial information loss when the adversarial camouflage texture shifts from the digital domain to the physical domain. The formula is expressed as follows:

(14) $${ℒ}_{NPS}=\sum_{p_{\mathrm{texture}}\in T_{adv}}\underset{c_{\mathrm{print}}\in C}{\min}|p_{\mathrm{texture}}-c_{\mathrm{print}}|,$$

$p_{\mathrm{texture}}$ denotes the pixel within the texture $T_{adv}$ and $c_{\mathrm{print}}$ denotes the color within a set of printable color C.

### 3.3. Physical Transformation

We adopt the method proposed by FCA for physical transformation to transform the rendered vehicle images into different environment scenes.

**Figure 2:**
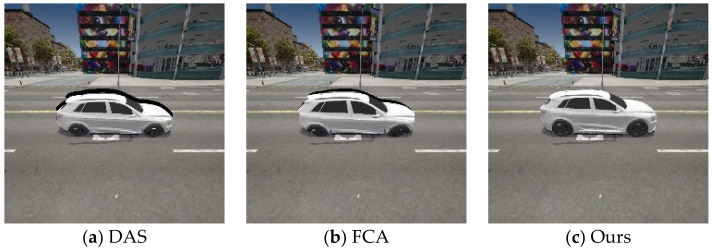
Comparison of transformation results of different methods.

However, although the data set collected at the sampling stage of the virtual engine that imitates the real world retains the position and orientation information, there is still a problem that the rendered images cannot fully cover the vehicles in the sampled images. Therefore, there are situations where there are abrupt black squares on the upper contour of the vehicle, and the lower contour is not fully displayed, resulting in a large gap between the images in the digital domain and the physical domain, as shown in Figure 2.

To address this issue, we referred to and improved the translation and scaling mentioned by MFA. We aligned and synthesized the rendered 2D images with the scene images, calculated the translation distance of the rendered images, limited the maximum translation distance, and minimized the black parts (with pixel values of 0) in the final synthesized image. The formula is expressed as follows:

(15) $$\begin{array}{c} x_{adv}=\Phi (I_{adv},m,x)=m\cdot T(I_{adv})+(1-m)\cdot x,\\ s.t.\;d\leq 10,\;B=\min\sum \left(255-x_{i,j}\right), \end{array}$$

𝒯 denotes the translation transformation of the rendered image $I_{adv}$, d indicates the translation distance of the rendered image, B represents the purely black portion within the image, and $x_{i,j}$ represents the pixel value at the i-th row and j-th column of the image. By minimizing the value of B, the pixel values of the black part within the image are reduced to the minimum.

## 4. Experiment

### 4.1. Experimental Setting

We conducted texture training on a single NVIDIA GeForce RTX 3090 graphics card, and the operating system is Windows 10.

#### 4.1.1. Evaluating Indicator

We utilized the attack success rate (ASR) proposed by Wu et al. [34] in 2020 as an indicator of the validity of adversarial textures, specifically, the percentage of the number of target vehicles that can be correctly detected without adding perturbations and those that cannot be correctly detected after adding perturbations.

(16) $$ASR=\frac{count[ℱ(x_{adv})\neq y]-count[ℱ(x)\neq y]}{count[ℱ(x)=y]}.$$

The second evaluation index is designated as P@0.5, which represents the precision of the model when the IoU threshold is 0.5. The calculation formula is

(17) $$P=\frac{TP}{TP+FP},$$

TP denotes the number of examples that are actually positive and detected as positive, and FP denotes the number of examples that are actually negative but detected as positive.

The evaluation indicators for the stealthiness effect are selected as the several indicators referred to in Section 2.3 of the previous text.

#### 4.1.2. Dataset

We opted to employ the identical data set as that of DAS, FCA, and MFA to train the adversarial textures and assess the attack effect of the adversarial textures. This dataset encompasses 12,500 training images and 3000 test images collected on the Carla simulation platform, along with the masks corresponding to the images, 3D vehicle models, the orientation and posture information corresponding to the vehicles, and camera lenses. Regarding the stealthiness of textures, we positioned the 3D vehicle model covered with the adversarial textures within the 3D scene and utilized the traditional rendering engine to render it to guarantee that the light, shadow, and material effects of the images utilized for testing the stealthiness are analogous to those tested in the real world.

#### 4.1.3. Implementation Details

We selected the conv1_1, conv2_1, conv3_1, conv4_1, and conv5_1 layers from the VGG19 network’s 16 convolutional layers to extract style features and used the conv4_2 layer to extract content features.

### 4.2. Digital Domain Experiment

**Figure 3:**
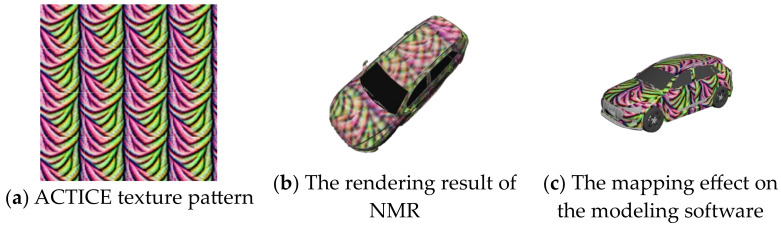
The texture pattern of ACTIVE and the rendering results on NMR.

To verify the effect of the method we proposed, we compared the attack effectiveness and stealthiness of several adversarial camouflage textures in the current field on different object detection models. Specifically, we compared no attack, random noise, DAS, FCA, DTA, and ACTIVE to validate the effectiveness of our textures. For the methods that have disclosed the training textures, such as FCA and DAS, we employed NMR to render them into the dataset scene through the transformation function based on the orientation and posture information. For the methods that have not disclosed the training textures, such as DTA and ACTIVE, we make use of the script provided by NMR to optimize the textures and minimize the disparity between the rendered model image and the reference image by adjusting the texture parameters of the model and generate the full-coverage camouflage textures to participate in the tests of the same test set, as shown in Figure 3.

**Figure 4:**
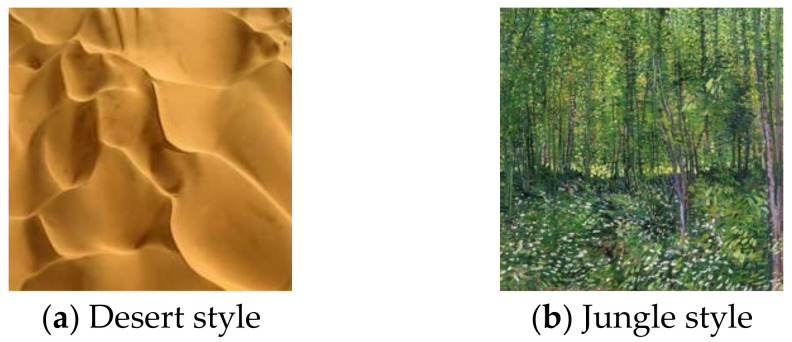
Two styles of images for adversarial texture style transfer.

The style images are selected as desert and jungle. The style images that participate in the texture training are shown in Figure 4.

#### 4.2.1. Effectiveness of Attack

Table 1 and Table 2, respectively, exhibit the ASR and P@0.5 of different textures under several classical object detection models in the digital world. Bold indicates the optimal value.

From the detection results presented in Table 1 and Table 2, it is evident that while our method’s performance in ASR and declined P@0.5 is marginally inferior to that of FCA, it still surpasses other attack methods. This can be attributed to our method’s optimization of texture through the incorporation of style loss in its computation, resulting in a slight reduction in attack performance. Nonetheless, this also demonstrates the robustness and transferability of our method, enabling successful attacks on diverse object detectors.

#### 4.2.2. Stealthiness of Attack

**Figure 5:**
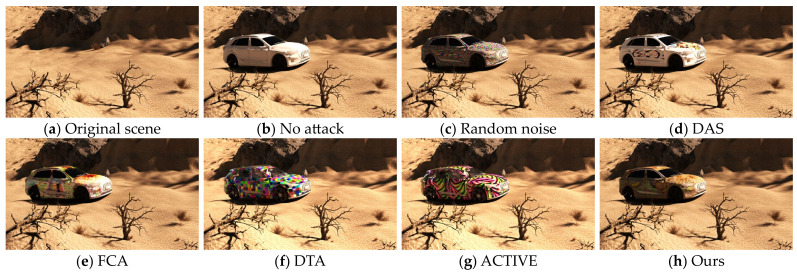
Comparison of rendered images with different textures in the desert scene in 3D modeling software.

**Figure 6:**
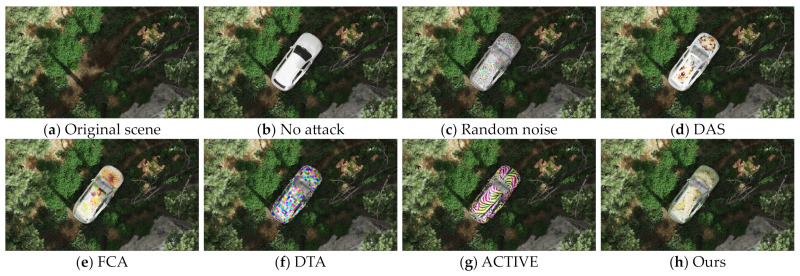
Comparison of rendered images with different textures in the jungle scene in 3D modeling software.

We covered the trained textures and other textures on the vehicle model, and in 3DSMAX2023 (PC-based 3D modeling rendering and production software), the model is placed in the specified scene and simulated the real-world light and shadow rendering as an image. Figure 5 and Figure 6 show the final rendering of these textures.

Table 3 and Table 4 show the SSIM, PSNR, and LIPIS of our method in different scenes, which can evaluate the stealthiness and visual naturalness of our adversarial camouflage textures.

By analyzing Table 3 and Table 4, our method has good stealthiness and visual naturalness compared to other adversarial textures in the specified scene. This can be attributed to the inclusion of a style transfer module in our training process, which extracts features from different layers of the style image to calculate style loss and adds pixel values of the style image to the adversarial texture. This allows our texture to retain aggressiveness while maintaining stealthiness and visual naturalness. When simulating adversarial attacks on a digital world simulation platform, compared to other different textures, our method has a higher attack success rate and a lower P@0.5 for the detection model (second only to FCA). Our method has the second-highest SSIM and PSNR and the lowest LPIPS when measuring the quality of adversarial texture rendering images and original scene images.

### 4.3. Physical Domain Experiment

**Figure 7:**
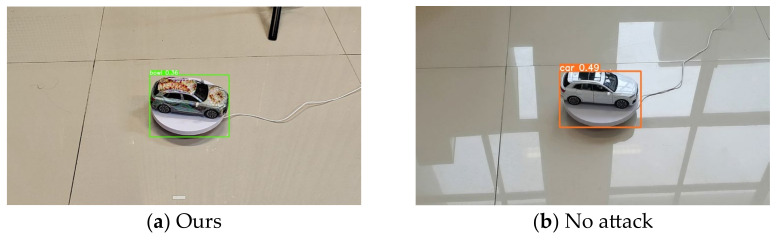
The attack effect of our method in the real world: (**a**) is predicted as a bowl, and (**b**) is predicted as a car.

**Figure 8:**
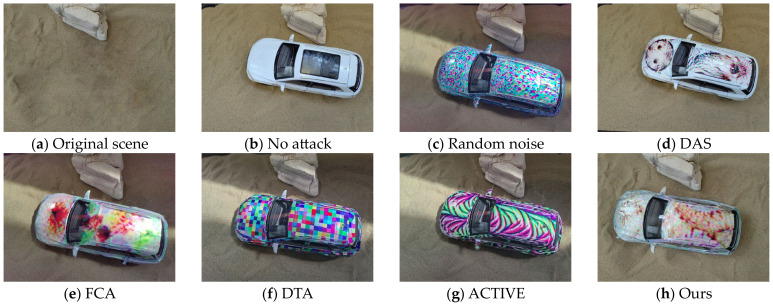
Comparison of shooting images with different textures in a desert scene in the real world.

**Figure 9:**
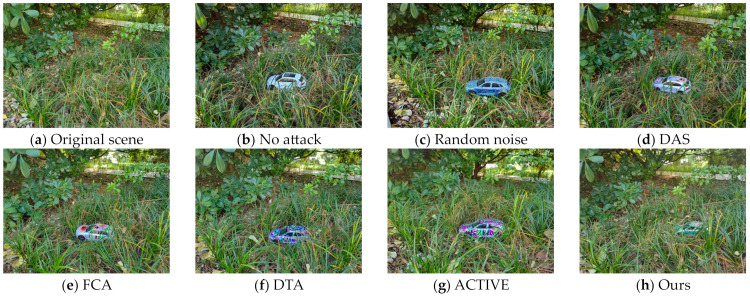
Comparison of shooting images with different textures in a jungle scene in the real world.

We use a large-format inkjet printer to print our adversarial camouflage textures and then cut and trim them. We then covered them to 1:24 scale Audi Q5 models to simulate the automotive coating appearance in the physical world. We conducted several sets of experiments on the textures mentioned in Section 4.2 to verify the actual effectiveness of our adversarial camouflage textures. Figure 7 shows the results of the attack in the physical world. Due to the difficulty in ensuring consistency of all elements except for camouflage before and after the attack, especially the slight shaking during shooting, it is difficult to ensure that all pixels in the captured image correspond one-to-one except for adversarial camouflage. Therefore, we reported the P@0.5 of the YOLOv5 model for attack effectiveness, LPIPS for adversarial examples, and original scenes for stealthiness. Figure 8 and Figure 9 show the vehicles participating in the specified scene of stealthiness testing in the real world. Table 5 shows the P@0.5 of different adversarial camouflage texture attack object detection models, while Table 6 and Table 7 show the LPIPS of different styles of adversarial camouflage textures in specified scenes.

It can be seen from Table 5 that our method also has certain attack effects in the real world, although this value may not accurately reflect the attacks received by the object detector in the real world because the value of P@0.5 only calculates the positive samples and negative samples when objects are detected, and the case where no objects are detected is not included in the calculation formula. And the environmental conditions of shooting in the real world cannot be controlled with the same precision as in the digital world. And by analyzing the detected images, we found that most attacks were successful when the object could not be detected rather than classified into other objects.

In the real-world experiment, it can be seen from Table 6 and Table 7 and Figure 8 and Figure 9 that our method has the lowest LPIPS value, and compared with other textures, our texture has good attack effectiveness and visual naturalness.

### 4.4. Ablation Study

In this section, we discussed the impact of setting style loss terms on texture attack effectiveness and stealthiness.

We removed the computation of adversarial loss and style loss when training textures and used the rendered images of these two textures to calculate the effectiveness and stealthiness of adversarial attacks in the digital world. We used several indicators mentioned in Section 4.2 for evaluation. The results are shown in Table 8 and Table 9.

We note from Table 6 and Table 7 that the inclusion of adversarial loss and style loss settings is essential for generating our proposed stealthy adversarial texture. Therefore, we integrate both loss terms into the calculation to optimize our final texture.

## 5. Conclusions

Due to the performance dependence of DNNs on training a large number of examples, in order to prevent such attacks, these adversarial examples can be included in the training set of the neural network for training, or the algorithm’s edge detection ability can be increased, as well as the ability to extract features from vehicles, car windows, and other parts that cannot cover the adversarial camouflage texture.

In this article, we improved the existing adversarial texture generation framework to generate adversarial camouflage textures with specified image styles. Specifically, we embed the style transfer module into the framework and calculate style loss, adversarial loss, smoothing loss, and NPS. A large number of experiments in both the digital simulation world and the real physical world have shown that our method has good attack effectiveness, stealthiness, and visual naturalness in specified scenes.

In our future research work, we will focus on adversarial example generation methods for a specific task (such as targeted attacks) and the application of discretization methods [35] to process data.

## Figures and Tables

**Table 1:** The comparison results of P@0.5 (%) in digital space.

	Detectors					
Methods	YOLOv5	YOLOv9	Faster R-CNN	SSD	DETR	Mean P@0.5
No attack	75.4	82.4	63.6	86.5	54.4	72.5
Random noise	62.5	68.2	55.8	78.8	40.6	61.2
FCA	**27.4**	**45.3**	**46.4**	75.3	**21.0**	**43.1**
DAS	66.1	69.7	61.7	83.9	45.0	65.3
DTA	72.1	75.9	63.9	80.0	46.2	67.6
ACTIVE	62.1	64.5	58.1	68.7	39.9	58.7
Ours—desert	47.6	55.2	56.9	**58.7**	31.7	50.0
Ours—jungle	47.8	55.5	57.1	59.9	34.2	50.9

**Table 2:** The comparison results of ASR (%) in digital space.

	Detectors					
Methods	YOLOv5	YOLOv9	Faster R-CNN	SSD	DETR	mASR
Random noise	19.8	21.2	26.8	38.5	42.3	29.7
FCA	**55.8**	**40.0**	**35.4**	54.6	**72.8**	**51.7**
DAS	16.5	0.8	2.0	6.6	31.4	11.5
DTA	0	7.1	0	14.3	24.5	9.2
ACTIVE	22.6	19.6	19.3	44.8	40.2	29.3
Ours—desert	41.1	35.2	24.5	68.5	58.4	45.5
Ours—jungle	37.8	34.5	21.6	**70.5**	54.6	43.8

**Table 3:** Comparison results of stealthiness of different textures in the desert scene in a simulated environment.

Indicator	No Attack	Random Noise	FCA	DAS	DTA	ACTIVE	Ours—Desert
SSIM	0.855	0.881	0.882	0.845	0.878	0.858	**0.883**
PSNR	14.560	15.894	15.230	14.778	15.314	15.626	**16.170**
LPIPS	0.128	0.093	0.100	0.126	0.141	0.127	**0.089**

**Table 4:** Comparison results of stealthiness of different textures in jungle scene in a simulated environment.

Indicator	No Attack	Random Noise	FCA	DAS	DTA	ACTIVE	Ours—Jungle
SSIM	**0.919**	0.911	0.913	0.913	0.913	0.913	0.914
PSNR	15.570	17.291	15.541	16.732	17.485	**18.147**	17.535
LPIPS	0.095	0.084	0.092	0.086	0.085	0.089	**0.082**

**Table 5:** Comparison of P@0.5 with different textures in the real world.

Methods	No Attack	Random Noise	DAS	FCA	DTA	ACTIVE	Ours—Desert	Ours—Jungle
P@0.5	87.5	78.9	83.9	77.8	72.8	49.3	**26.3**	**27.3**

**Table 6:** Comparison of LPIPS with different textures in real desert scenes.

Methods	No Attack	Random Noise	DAS	FCA	DTA	ACTIVE	Ours—Desert
LPIPS	0.488	0.582	0.522	0.6	0.616	0.623	**0.335**

**Table 7:** Comparison of LPIPS with different textures in real desert scenes.

Indicator	No Attack	Random Noise	DAS	FCA	DTA	ACTIVE	Ours—Jungle
LPIPS	0.488	0.496	0.487	0.474	0.716	0.513	**0.423**

**Table 8:** The P@0.5 (%) of the loss term ablation experiment.

	Detector		
Loss Term	YOLOv5	YOLOv9	SSD
No adv loss	54.6	65.6	60.6
No style loss	35.9	42.0	32.1

**Table 9:** Comparative results of the impact of loss terms on stealthiness.

Loss Term	SSIM	PSNR	LPIPS
No adv loss	0.923	20.109	0.056
No style loss	0.914	17.518	0.082

## Data Availability

Data related to the current study are available from the corresponding author upon reasonable request.
